# A comparison of ground reaction forces during level and cross-slope walking in Labrador Retrievers

**DOI:** 10.1186/s12917-014-0241-4

**Published:** 2014-09-28

**Authors:** Therese Strasser, Christian Peham, Barbara A Bockstahler

**Affiliations:** Department for Companion Animals and Horses, University Clinic for Small Animals, Small Animal Surgery, Section for Physical Therapy, University of Veterinary Medicine, Vienna, Austria; Department for Companion Animals and Horses, Equine University Clinic, Equine Surgery, Movement Science Group Vienna, University of Veterinary Medicine, Vienna, Austria

**Keywords:** Cross-slope, Dog, Gait analysis, Ground reaction forces, Pressure plate

## Abstract

**Background:**

Inclined or slippery surfaces and various other types of obstacles are common demands in our environment. Dogs with impaired locomotion might have difficulties to manage rough terrain. Gait analyses using force plates or pressure plates, which are well established to characterize limb loads in human medicine as well as in animals, are mostly limited to level surfaces. Therefore, the aim of this study was to investigate the effect of cross-slope walking in ten healthy Labrador Retrievers using a pressure plate walkway system. The dogs walked over the pressure plate on a level surface, with a lateral elevation angle of 10° (CS1) or 15° (CS2) until five valid trials were achieved. Three measurements were obtained at weekly intervals. Peak vertical force (PFz), vertical impulse (IFz), step length, and velocity were determined.

**Results:**

Compared to level walking (LW), cross-slope walking was associated with a significant decrease in GRF of the up-slope (US) hindlimb, which was compensated for by the down-slope (DS) forelimb. The other diagonal limb pair showed less pronounced effects during CS1, but in CS2 more weight was shifted onto the DS hindlimb during the first two measurements, thus reducing weight on the US forelimb (for IFz). The effect diminished from trial to trial, with GRF values approaching LW standards finally. The IFz was a more sensitive measure than the PFz. The step length of the DS forelimb was significantly decreased in both cross-slope conditions, while the step length of the US forelimb only decreased during CS2.

**Conclusions:**

The dogs adapted their gait pattern and step length to compensate for the discrepancy in apparent leg length caused by the cross-slope. The results suggest that cross-slope walking requires functional musculoskeletal adaptations that may be difficult for animals with impaired locomotion. Further, this knowledge might be of clinical impact for early diagnosis of neurological disorders, mild lameness and proprioceptive deficits.

## Background

Measuring ground reaction forces (GRF) is a common method to describe gait symmetry and to evaluate limb loading in humans as well as in dogs. Most studies have been performed either on a treadmill or using force plates, usually mounted in the ground [1]. Also the use of pressure plates is a valid method like shown by Lascelles and Oosterlinck as well in dogs as in horses [2-4]. Lascelles et al. [2] showed that absolute GRF values determined with the use of a pressure-sensitive walkway were lower than those reported in studies using a force plate, but with a similar trend, suggesting that both methods are suitable for use in comparative studies. Oosterlinck et al. [3] determined the accuracy of pressure plate kinetic asymmetry indices and their correlation with the scores of visual gait assessment in dogs with unilateral hindlimb lameness. They showed that the asymmetry indices of the Peak vertical force (PFz) and vertical impulse (IFz) measured using a pressure plate were reliable indicators of clinical lameness in dogs. Further, Oosterlinck et al. [3] found out that the paw contact area is a very sensitive variable for evaluating limb loading symmetry. Souza et al. [5] found that the vertical forces in the pads of dogs suffering from cranial cruciate ligament rupture were lower in the affected limb and they showed a compensatory effect in the forelimb and contralateral hindlimb pads.

Gait analyses provide useful information on normal and disturbed limb loads and have frequently been used in basic research and for evaluating treatment outcomes [6-8]. The results from these studies provide information crucial to our understanding of canine locomotion. However, in everyday life, pedestrians rarely walk on completely flat surfaces and frequently encounter inclined surfaces, including rough terrain and man-made slopes such as tilted sidewalks [9]. Moreover, humans and animals have to contend with various types of obstacles during locomotion, such as stones, roots, and other kinds of unevenness [10]. These natural and artificial circumstances do not usually pose a challenge to orthopedically and neurologically sound humans and animals that are able to adapt their gait accordingly. However, the asymmetrical demands of walking on uneven ground may introduce functional musculoskeletal and balance barriers, such as seen in humans when cross-slope walking [9]. In such circumstances, the functional leg length can be altered by shortening the up-slope (US) leg while elongating the down-slope (DS) leg, resulting in a reduced step width and an increase in mediolateral GRF [9].

As canines live in the same physical environment as humans and encounter the same ground, it is likely that they make comparable gait adaptions. As with afflicted humans, dogs suffering from neurological disorders often have severe problems walking on uneven or slippery surfaces. Animals with a disturbed gait due to orthopedic problems that lead to pain and/or altered joint biomechanics might also have difficulties managing rough terrain.

There have been only a few studies of canines walking on inclines and declines. Lauer et al. [11] investigated the hamstring, gluteal and quadriceps muscle activity and the range of motion (ROM) of the hip and stifle joint during incline and decline walking and found that walking on an incline increases hamstring activity with only a minimal effect on joint kinematics. However, Millard et al*.* [12] found significantly greater ROMs of the hip, stifle and tarsal joints when decline walking was performed on stairs. In contrast, the ROM in the forelimbs of dogs walking an incline was found to be significantly greater than when walking up stairs [13].

Compared to dogs, humans use a greater overall ROM in the hip and stifle joint when walking on level surfaces and on stairs, though the ROM of the tarsal joint is greater in dogs [14]. A kinematic analysis of joint biomechanics during uphill and downhill walking and walking over an obstacle by Holler et al*.* [15] showed that uphill walking caused increased hip joint flexion and decreased stifle joint flexion compared to downhill walking. To date, however, there have not been any comparable studies on GRF during cross-slope walking in dogs. It could be expected that the DS limbs have larger GRFs than the US limbs but this prediction has not previously been addressed. Therefore, the aim of this study was to perform a detailed examination of canine cross-slope locomotion to provide information on GRF and step length in both hind- and forelimbs.

## Methods

### Animals

The study was approved by the Institutional Ethics Committee in accordance with guidelines for good scientific practice and with national legislation (10/09/97/2011).

The study included ten client-owned, adult, clinically sound Labrador Retrievers between one to seven years of age (3.4 ± 2.3 y) and from 24.0 to 36.0 kg (27.7 ± 3.5 kg). All dogs underwent an orthopedic and neurological examination of all four legs and the vertebral column. Dogs were only included if neither a visible lameness nor pain during manipulation of the joints was detectable.

### Equipment

A pressure plate developed by Zebris (FDM Type 2, Zebris Medical GmbH, Allgäu, Germany) covered with a rubber layer was mounted in the middle of a 7 m runway. The pressure plate had a measurement area of 203.2 × 54.2 cm and included 15360 sensors with a sampling rate of 100 Hz. The plate was covered with a rubber mat to hide the measuring area from the dogs’ sight and prevent slipping. For cross-slope walking, the plate was elevated on the left-hand side (walking direction) to an elevation of 10° and 15° using wooden wedges. The trials were video recorded using a Panasonic NV-MX500. Data were recorded using WinFDM software (v1.2.2; Zebris Medical GmbH).

### Study design

The dogs were given sufficient time to acclimate to the measurement area and to get accustomed to the equipment by walking freely in the room and over the plate. Subsequently they were walked over the plate on the leash a few times until they showed a smooth and harmonious gait pattern. This procedure was repeated before each of the measurements conditions. The dogs were walked at their own comfortable speed over the platform by their owners under three different conditions: level walking (LW), cross-slope walking with a lateral elevation of 10° (CS1), and cross-slope walking with a lateral elevation of 15° (CS2). The dogs walked in the same direction in each trial, with the handler on their right-hand side. Trials were accepted as valid if the dog walked in a straight line with the head forward over the plate without apparent change of velocity and further by means of uniform of the GRF data. In addition, trials under cross-slope conditions were excluded if one of the animal’s paws slipped on the plate, which was identified based on the monitoring during the measure and also by the shape of the GRF curves. The dogs were walked over the pressure plate until five valid trials for each condition were achieved [6,7,12,15]. Depending on the dogs’ size and step length, one to two full motion cycles per pass over the plate could have been evaluated, resulting in a minimum number of seven steps during LW and five steps each during CS1 and CS2. Measurements were collected once a week for a total of three measurements. Pressure prints of the footfalls were manually identified from the video recordings and matched with the corresponding limbs. To eliminate measurement error due to the stepping on the plate in the evaluated conditions for cross-slope conditions, the initial footfalls on the plate were excluded so that the evaluation started with the second motion cycle. The final contact between the hindlimb and the elevated pressure plate was also excluded. The left paws were designated as US limbs, and the right paws were designated as DS limbs.

### Data analysis

Data were processed using specially developed software (Pressure Analyzer 1.3.0.2, Michael Schwanda). From each step, the mean PFz, IFz and step length were calculated for each condition and measurement day. The velocity of the dogs was calculated for the US forelimb based on the time between successive US forelimb ground contacts, and only trials with a velocity of 0.9–1.1 m/s were accepted.

To assess the compensatory effects of cross-slope walking, the force data were normalized to the sum of all forces exerted by the four limbs and expressed as % total force (TF).

### Statistical analysis (SPSS, version 22)

A Kolmogorov-Smirnov test was used to evaluate data distributions. All data were normal distributed. Analysis of variance (ANOVA) for repeated measurements was performed to investigate differences between LW, CS1 and CS2, and the first and the following two measurements. Data are expressed as the mean ± standard deviation, and a *P* ≤ 0.05 was considered as statistically significant.

## Results

**Figure 1:**
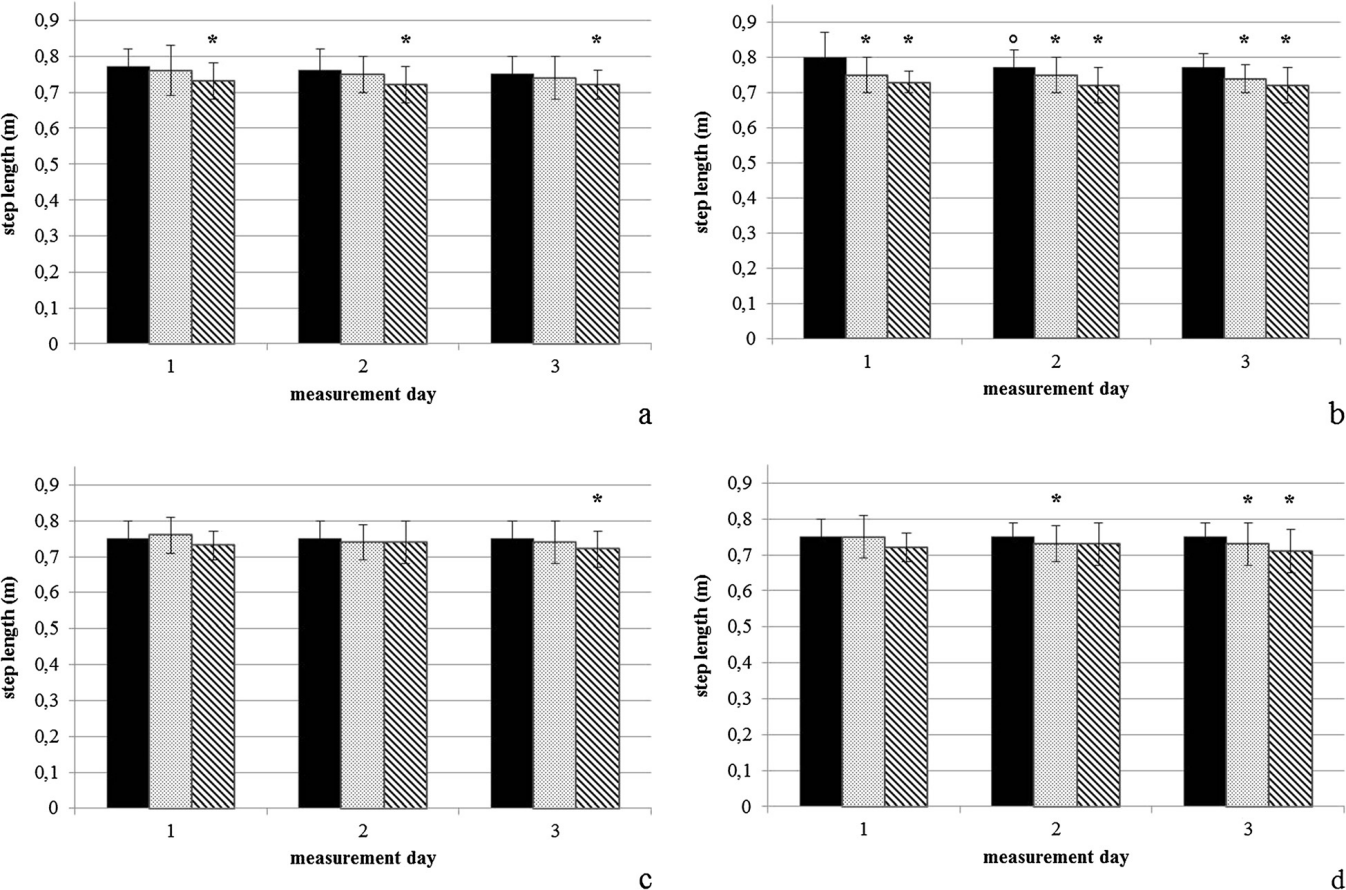
**Step length (m). (a)** Up-slope [US] forelimb, **(b)** down-slope [DS] forelimb, **(c)** US hindlimb, **(d)** DS hindlimb. The black bars represent level walking, the dotted bars indicate cross-slope 1, and the shaded bars indicate cross-slope 2. Data are expressed as mean ± standard deviation. *represents significant differences compared to Level walking, °represents significant differences compared to the first measurement.

**Figure 2:**
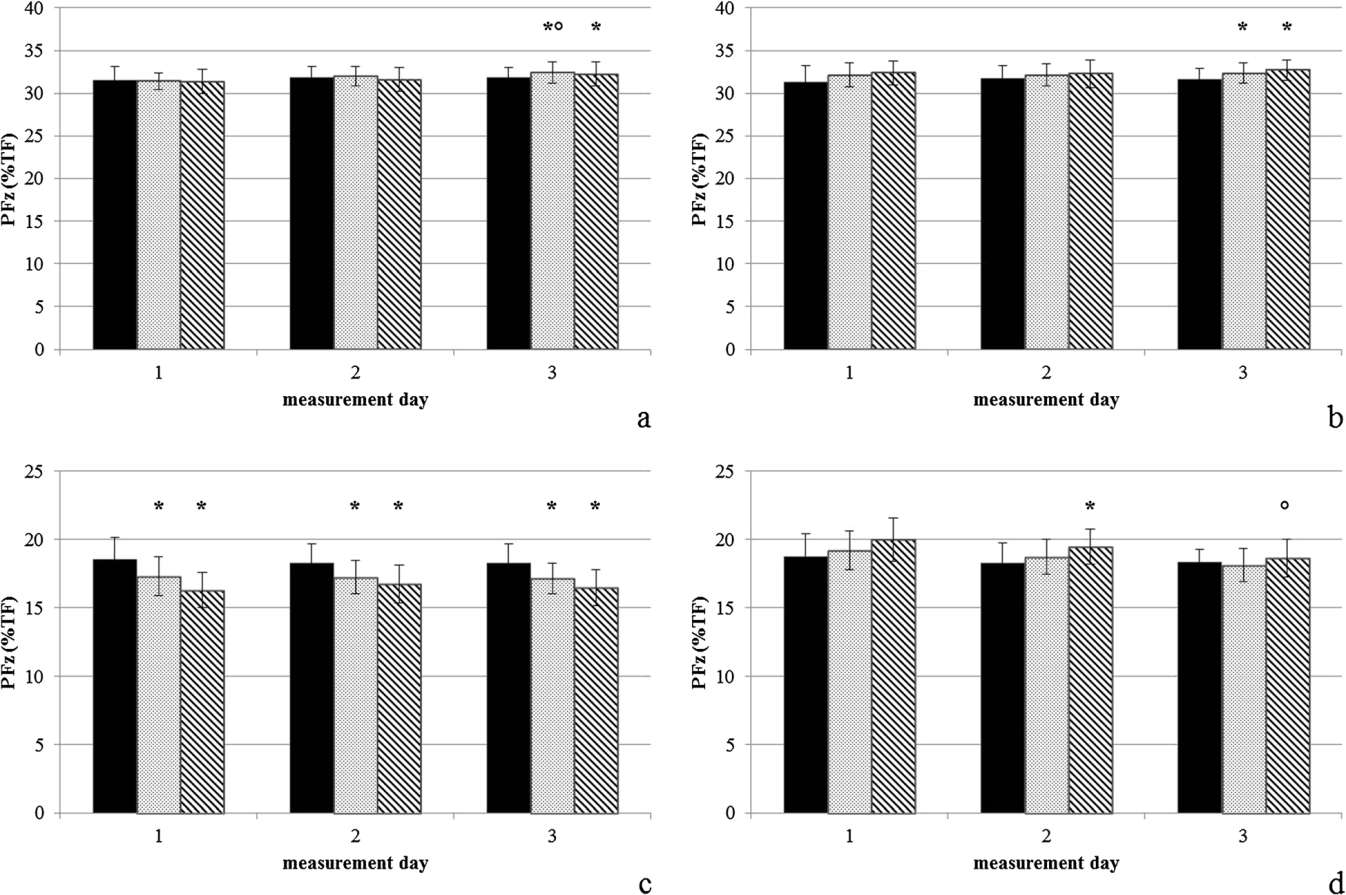
**Peak vertical force (% total force). (a)** Up-slope [US] forelimb, **(b)** down-slope [DS] forelimb, **(c)** US hindlimb, **(d)** DS hindlimb. The black bars represent level walking, the dotted bars indicate cross-slope 1, and the shaded bars indicate cross-slope 2. Data are expressed as mean ± standard deviation. *represents significant differences compared to Level walking, °represents significant differences compared to first measurement.

**Figure 3:**
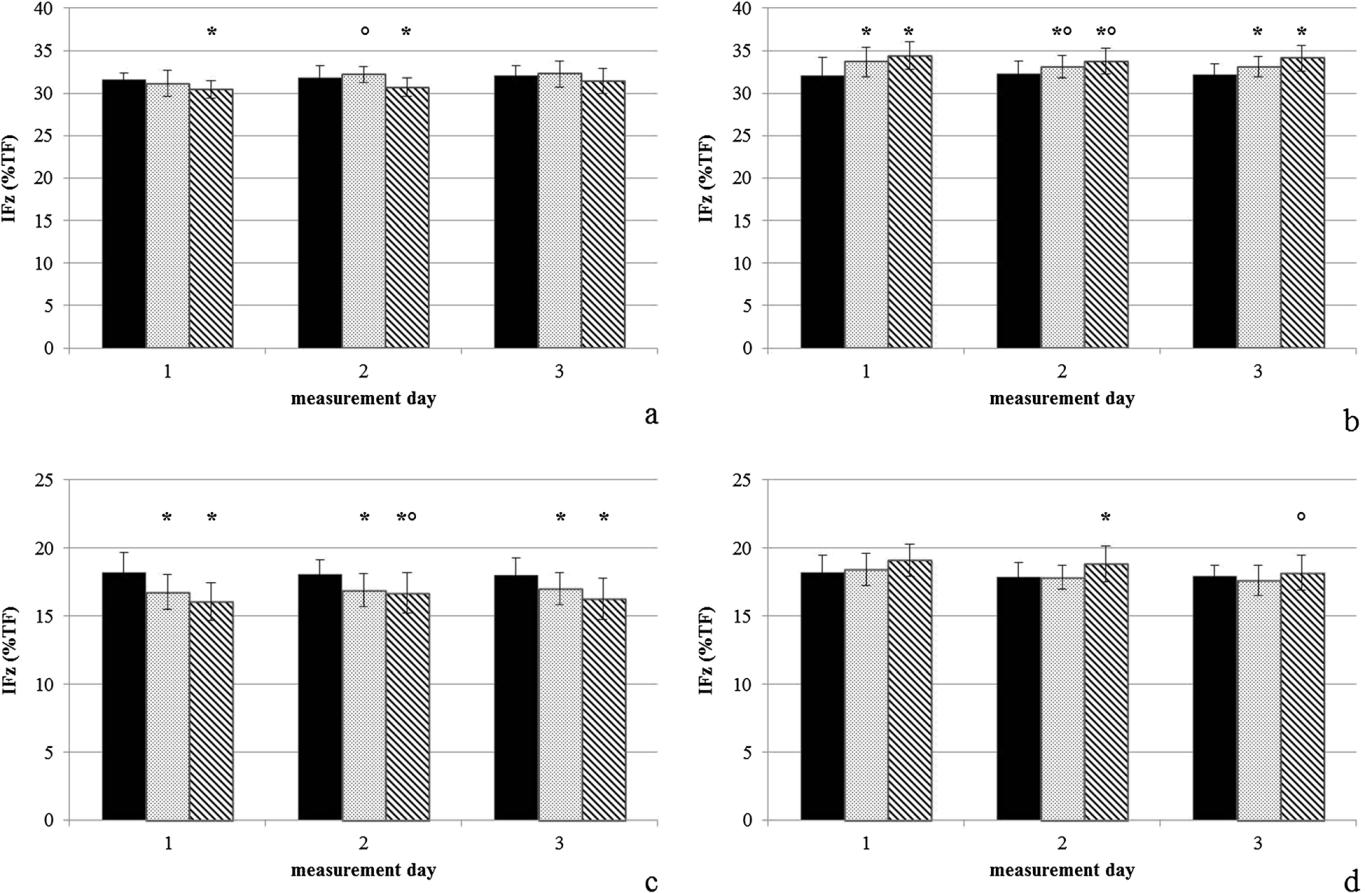
**Vertical impulse (% total force). (a)** Up-slope [US] forelimb, **(b)** down-slope [DS] forelimb, **(c)** US hindlimb, **(d)** DS hindlimb. The black bars represent level walking, the dotted bars indicate cross-slope 1, and the shaded bars indicate cross-slope 2. Data are expressed as mean ± standard deviation. *represents significant differences compared to Level walking, °represents significant differences compared to first measurement.

Mean values and standard deviation for Peak Vertical Force (PFz), Vertical Impulse (IFz) and step length are given in Table 1. Differences between first measurement (M1) and following measurements refer to the respective exercise. Differences in step length, PFz and IFz are illustrated in Figures 1, 2 and 3.

**Table 1:** **Step length and forces with cross-slope walking in dogs**

				**M1**	**M2**	**M3**
**Step length (m)**	**LW**	US	forelimb	0.77 ± 0.05	0.76 ± 0.06	0.75 ± 0.05
			hindlimb	0.75 ± 0.05	0.75 ± 0.05	0.75 ± 0.05
		DS	forelimb	0.80 ± 0.07	0.77 ± 0.05^b^	0.77 ± 0.04
			hindlimb	0.75 ± 0.05	0.75 ± 0.04	0.75 ± 0.04
	**CS1**	US	forelimb	0.76 ± 0.07	0.75 ± 0.05	0.74 ± 0.06
			hindlimb	0.76 ± 0.05	0.74 ± 0.05	0.74 ± 0.06
		DS	forelimb	0.75 ± 0.05^a^	0.75 ± 0.05^a^	0.74 ± 0.04^a^
			hindlimb	0.75 ± 0.06	0.73 ± 0.05^a^	0.73 ± 0.06^a^
	**CS2**	US	forelimb	0.73 ± 0.05^a^	0.72 ± 0.05^a^	0.72 ± 0.04^a^
			hindlimb	0.73 ± 0.04	0.74 ± 0.06	0.72 ± 0.05^a^
		DS	forelimb	0.73 ± 0.03^a^	0.72 ± 0.05^a^	0.72 ± 0.05^a^
			hindlimb	0.72 ± 0.04	0.73 ± 0.06	0.71 ± 0.06^a^
**PFz (%TF)**	**LW**	US	forelimb	31.46 ± 1.56	31.78 ± 1.35	31.85 ± 1.08
			hindlimb	18.53 ± 1.64	18.28 ± 1.44	18.26 ± 1.37
		DS	forelimb	31.29 ± 1.86	31.67 ± 1.52	31.60 ± 1.26
			hindlimb	18.73 ± 1.75	18.27 ± 1.53	18.30 ± 1.03
	**CS1**	US	forelimb	31.39 ± 1.04	31.96 ± 1.13	32.40 ± 1.16^a, b^
			hindlimb	17.31 ± 1.45^a^	17.24 ± 1.19^a^	17.15 ± 1.09^a^
		DS	forelimb	32.10 ± 1.41	32.10 ± 1.29	32.33 ± 1.18^a^
			hindlimb	19.21 ± 1.35	18.72 ± 1.33	18.12 ± 1.16
	**CS2**	US	forelimb	31.35 ± 1.40	31.55 ± 1.37	32.22 ± 1.44^b^
			hindlimb	16.28 ± 1.31^a^	16.74 ± 1.40^a^	16.47 ± 1.28^a^
		DS	forelimb	32.37 ± 1.44	32.26 ± 1.61	32.71 ± 1.18^a^
			hindlimb	20.00 ± 1.61	19.46 ± 1.29^a^	18.61 ± 1.38^b^
**IFz (%TF)**	**LW**	US	forelimb	31.58 ± 0.79	31.82 ± 1.45	31.98 ± 1.18
			hindlimb	18.16 ± 1.49	18.05 ± 1.11	17.97 ± 1.26
		DS	forelimb	32.06 ± 2.14	32.27 ± 1.46	32.14 ± 1.30
			hindlimb	18.20 ± 1.26	17.85 ± 1.09	17.91 ± 0.82
	**CS1**	US	forelimb	31.13 ± 1.54	32.18 ± 0.93^b^	32.27 ± 1.46
			hindlimb	16.76 ± 1.28^a^	16.90 ± 1.21^a^	16.99 ± 1.25^a^
		DS	forelimb	33.67 ± 1.67^a^	33.08 ± 1.30^a, b^	33.12 ± 1.24^a^
			hindlimb	18.44 ± 1.19	17.85 ± 0.89	17.62 ± 1.13
	**CS2**	US	forelimb	30.45 ± 1.02^a^	30.73 ± 1.11^a^	31.44 ± 1.49
			hindlimb	16.04 ± 1.40^a^	16.70 ± 1.47^a, b^	16.27 ± 1.49^a^
		DS	forelimb	34.40 ± 1.64^a^	33.73 ± 1.55^a, b^	34.12 ± 1.52^a^
			hindlimb	19.11 ± 1.21	18.84 ± 1.35^a^	18.18 ± 1.26^b^

*Abbreviations*: *CS1* 10° cross-slope; *CS2* 15° cross-slope; *DS* down-slope; *IFz* vertical impulse; *LW* level walking; *M1* first measurement; *M2* second measurement; *M3* third measurement; *PFz* peak vertical force; *TF* total force; *US* up-slope. Values are expressed as mean ± standard deviation, ^a^ *P* < 0.05 *vs.* LW; ^b^ *P* < 0.05 *vs.* M1.

### LW

The average LW velocity of the US frontlimb was 1.06 ± 0.1 m/s in M1, 1.09 ± 0.1 m/s in the second measurement (M2) and 1.03 ± 0.1 m/s in the third measurement (M3), which were not significantly different. There were no differences in PFz or IFz from any of the limbs between trials during LW. However, the DS forelimb showed a significantly shorter step length in M2 compared to M1 (P = 0.053).

### Differences of the US forelimb compared to LW

#### CS1

No significant changes in step length between trials or compared to LW were found. However, during CS1 the PFz in the US forelimb was significantly higher in M3 compared to M1 (P = 0.031) and compared to LW (P = 0.008). The IFz of the US forelimb in M2 was significantly increased compared to M1 (P = 0.030), but was not different from LW during CS1.

#### CS2

The step lengths of the US forelimb were similar between CS2 trials, but were significantly shorter than for LW (P = 0.003 for M1; P = 0.014 for M2; P = 0.010 for M3). The PFz remained unchanged compared to LW but there was a significant increase from M1 to M3 (P = 0.006). The IFz in the US forelimb was significantly decreased in M1 and M2 compared to LW (P = 0.013 for M1; P = 0.021 for M2).

### Differences of the US hindlimb compared to LW

#### CS1

The step length in the US hindlimb did not differ from LW during CS1, or between trials. However, significantly lower PFz and IFz values were observed in all three trials compared to LW (PFz: P = 0.006 for M1; P = 0.002 for M2; P = 0.005 for M3; IFz: P = 0.005 for M1; P = 0.000 for M2; P = 0.021 for M3).

#### CS2

In M3, the US hindlimb showed a significantly decreased step length compared to LW (P = 0.041). As in CS1, there were consistently significantly lower values for PFz and IFz during CS2 compared to LW (PFz: P = 0.000 for M1; P = 0.003 for M2; P = 0.003 for M3; IFz: P = 0.000 for M1; P = 0.002 for M2; P = 0.006 for M3), though IFz was significantly higher in M2 than in M1 (P = 0.053).

### Differences of the DS forelimb compared to LW

#### CS1

The step length of the DS forelimb was significantly shorter in all three trials compared to LW (P = 0.013 for M1; P = 0.046 for M2; P = 0.001 for M3). While the PFz of the DS forelimb was only significantly increased in M3 (P = 0.020), the IFz values were increased in all three trials compared to LW (P = 0.002 for M1; P = 0.023 for M2; P = 0.016 for M3), though the M2 IFz was significantly lower than M1 (P = 0.040).

#### CS2

The step length of the DS forelimb was also significantly shorter in all three trials during CS2 compared to LW (P = 0.007 for M1; P = 0.004 for M2; P = 0.000 for M3). Similar to what was observed in CS1, the PFz in CS2 was significantly higher in M3 compared to LW (P = 0.012), and the IFz values were increased in all three trials compared to LW (P = 0.001 for M1; P = 0.002 for M2; P = 0.004 for M3), with a significantly lower IFz in M2 compared to M1 (P = 0.044).

### Differences of the DS hindlimb compared to LW

#### CS1

The step length in the DS hindlimb during CS1 was significantly shorter than in LW during M2 (P = 0.007) and M3 (P = 0.037). Neither of the GRF parameters indicated any differences for the DS hindlimb.

#### CS2

Compared to LW, the step length of the DS hindlimb became shorter during CS2, with a significant difference observed in M3 (P = 0.010). The PFz and IFz values decreased over the trials, showing a significant difference at M3 vs. M1 (PFz: P = 0.001; IFz: P = 0.005), however both values in the DS hindlimb were higher in M2 compared to LW (PFz: P = 0.015; IFz: P = 0.028).

## Discussion

The results of this study indicate that dogs show significant asymmetrical compensations between the US and DS paws when cross-slope walking, similar to what has been shown in humans [9]. These compensations became more apparent in CS2. As a compensation mechanism of the decreased GRFs of the US-hindlimb, GRFs are not only increased in the contralateral forelimb but in parts also in the contralateral DS hindlimb (M2). IFz showed more changed values, especially in the forelimbs, than the PFz. Accordingly the IFz was a more sensitive measure than the PFz, possibly due to the fact that the IFz represents the events during the whole stance phase when the dog is compensating the cross-slope, while the PFz indicates only a single point on the GRF curve when the entire kinetic energy encounters the ground. The other diagonal limb pair, the DS hindlimb and US forelimb, did not show pronounced effects during CS1 (with the exception of the PFz in M3) but changed between measurements for CS2. Initially, during M1, the animals shifted more weight onto the DS hindlimb and reduced the load on the US forelimb (for IFz), but in subsequent trials the GRF values increasingly approached those seen in LW. This suggests a general habituation to cross-slope walking over time. In summary, the dogs modified their kinetics together with the step length to compensate for the apparent discrepancy in leg length caused by the slope. A comparable mechanism has been described in humans for maintaining locomotion and vertical balance and to prevent falling [9,10].

Like in our study, frequently a step number of at least five [6,7] is used, but it should be considered that a higher number of steps and testing the reliability under different circumstances should be done to clarify the habituation effects more in detail.

One influence factor on our results is that pressure plates can only register vertical forces. At our measured angles, the forces acting on the plate are oblique to the surface, thus also creating horizontal forces which are not captured by the sensors. This measurement error must be considered by interpreting the results. It might be of interest to determine the paw contact area during LW and under cross-slope condition to describe the touchdown of the paws in detail.

Evaluation of joints kinematics in human studies indicates that there are major changes in the behavior of the joints. It is presumed that these changes also occur in dogs during cross-slope walking, which will be investigated in follow-up studies. In 2002, the redistribution of foot pressure during side-slope walking in humans was described [16]. The results showed that the pressures increased depending on the relative position of the foot on the slope. Therefore, it would also be interesting to investigate the distribution of pressure within the paws.

In this study the contact area of the paws was not considered as a variable, but as shown by Oosterlinck et al. this parameter could be used as an additional variable to describe the limb-loading symmetry [3]. In subsequent study this parameter should be also evaluated under different conditions, for example in lame and non-lame dogs absolving the exercises described in this paper.

In addition, an investigation of muscle activity patterns will help to complete our understanding of the biomechanical adaptions during cross-slope walking.

A study by Colborne [17] suggested the possibility for a “right hindlimb dominancy” in one dog, which could influence the results of the present study as the dogs were always led in the same direction over the plate (with the left-side US). Alternating the direction of the animals in future studies will help to test Colborne’s idea and provide additional information concerning the familiarization effect detected in our study. The present study was also restricted to healthy Labrador Retrievers that were easily able to handle cross-slope walking, a task that would likely be difficult for orthopedically/neurologically impaired animals. We assume that animals suffering from neurological disorders might have more difficulties to manage cross-slope walking and also mild lameness and proprioceptive deficits might be more obvious than during level walking. Accordingly, some of these dysfunctions might be detected earlier using this kind of pressure plate analysis. Moreover, a prolonged exposure to conditions that induce different leg lengths, such as after femoral head and neck excision, may exacerbate other musculoskeletal disorders. It should also be noted that no geriatric animals were included in the present study. As it is known that elderly people have problems adapting to uneven and/or slippery surfaces [18], future studies should investigate whether comparable problems occur in senior dogs.

This kind of analysis might also be useful in the development of rehabilitation programs and to screen the progress of therapies.

## Conclusions

The results of this study indicate that cross-slope walking requires substantial adaptions of the GRF to permit undisturbed locomotion, hold vertical balance and to prevent falling. These adaptations may be difficult for geriatric animals, or those with orthopedic or neurological disorders. The results contribute to the understanding of canine biomechanics and will be useful in the early diagnosis of neurological and orthopedic disorders, the development of rehabilitation and prevention programs for animals with impaired locomotion and the screening of therapy progress.

## Abbreviations

LW: Level walking
CS1: Cross-slope 10°
CS2: Cross-slope 15°
PFz: Peak vertical force
IFz: Vertical impulse
US: Up-slope
DS: Down-slope
GRF: Ground reaction forces
ROM: Range of motion
TF: Total force
M1: First measurement
M2: Second measurement
M3: Third measurement.
